# Supplementary material for: Deep-learning-based segmentation using individual patient data on prostate cancer radiation therapy
Source: PLoS One. 2024 Jul 31;19(7):e0308181. doi: 10.1371/journal.pone.0308181 (PMC11290636; doi:10.1371/journal.pone.0308181)
Supplement: Attachment — Submitted filename: Response_Reviewers_20240607-1.docx [file pone.0308181.s001.docx]

PONE-D-23-40530

Deep-learning-based segmentation using individual patient data of prostate cancer radiation therapy.

PLOS ONE

**Journal Requirements:**

1. When submitting your revision, we need you to address these additional requirements.

Please ensure that your manuscript meets **PLOS ONE's style requirements**, including those for file naming. The PLOS ONE style templates can be found at 
https://journals.plos.org/plosone/s/file?id=wjVg/PLOSOne_formatting_sample_main_body.pdf and 
https://journals.plos.org/plosone/s/file?id=ba62/PLOSOne_formatting_sample_title_authors_affiliations.pdf

**🡪**: It was modified to PLOS ONE's style through English proofreading.

2. Please note that PLOS ONE has **specific guidelines on code sharing for submissions** in which author-generated code underpins the findings in the manuscript. In these cases, all author-generated code must be made available without restrictions upon publication of the work. Please review our guidelines at https://journals.plos.org/plosone/s/materials-and-software-sharing#loc-sharing-code and ensure that your code is shared in a way that follows best practice and facilitates reproducibility and reuse.

**🡪**: We're constantly revising code sharing (<https://github.com/SangWoonJeong/DVF-based-segmentation>) to validate guidelines and promote reproducibility.

3. We note that the grant information you provided in the **‘Funding Information’ and ‘Financial Disclosure’ sections do not match.**

When you resubmit, please ensure that you provide the correct grant numbers for the awards you received for your study in the ‘Funding Information’ section.

**🡪 The grant numbers were corrected.**

4. Thank you for stating the following in the **Acknowledgments Section** of your manuscript: 
This research was supported by the National Research Foundation of Korea grants funded by the Korean government (MSIT) (2022R1A2C2004694 and 2021M28A1048108).

We note that you have provided funding information that is not currently declared in your Funding Statement. However, funding information should not appear in the Acknowledgments section or other areas of your manuscript. We will only publish funding information present in the Funding Statement section of the online submission form. 

Please remove any funding-related text from the manuscript and let us know how you would like to update your Funding Statement. Currently, your Funding Statement reads as follows: 
@ Initials of the authors who received each award: Y.Han
@ Grant numbers: 2022R1A2C2004694 and 2021M2E8A1048108
@The full name of each funder:  National Research Foundation of Korea grants funded by the Korean government (MSIT)
@URL of each funder website: https://www.nrf.re.kr/eng/index
@Did the sponsors or funders play any role in the study design, data collection and analysis, decision to publish, or preparation of the manuscript?: No

Please include your amended statements within your cover letter; we will change the online submission form on your behalf.

🡪 Thank you. The stated funding information is correct. I will include that information in the cover letter.

5. Thank you for uploading your study's underlying data set. Unfortunately, the repository you have noted in your Data Availability statement does not qualify as an acceptable data repository according to PLOS's standards.

At this time, please upload the minimal data set necessary to replicate your study's findings to a stable, public repository (such as figshare or Dryad) and provide us with the relevant URLs, DOIs, or accession numbers that may be used to access these data. For a list of recommended repositories and additional information on PLOS standards for data deposition, please see <https://journals.plos.org/plosone/s/recommended-repositories>

**🡪**: We shared the code used in the study (at <https://github.com/SangWoonJeong/DVF-based-segmentation>), but not the data. Data sharing must be approved through the hospital's data review committee. The data sharing request was applied, and the review is under process. Once data sharing is approved, we will share it to the recommended repository.

6. When completing the data availability statement of the submission form, you indicated that you will make your data available on acceptance. We strongly recommend all authors decide on a data sharing plan before acceptance, as the process can be lengthy and hold up publication timelines. Please note that, though access restrictions are acceptable now, your entire data will need to be made freely accessible if your manuscript is accepted for publication. This policy applies to all data except where public deposition would breach compliance with the protocol approved by your research ethics board. If you are unable to adhere to our open data policy, please kindly revise your statement to explain your reasoning and we will seek the editor's input on an exemption. Please be assured that, once you have provided your new statement, the assessment of your exemption will not hold up the peer review process.

**🡪**: To share the image data used in this research, data open must be approved after evaluating images by the hospital's data review committee. The evaluation process takes from one to three months. Therefore, patient image data sharing will be possible after permission.

7. Please review your reference list to ensure that it is complete and correct. If you have cited papers that have been retracted, please include the rationale for doing so in the manuscript text or remove these references and replace them with relevant current references. Any changes to the reference list should be mentioned in the rebuttal letter that accompanies your revised manuscript. If you need to cite a retracted article, indicate the article’s retracted status in the References list and also include a citation and full reference for the retraction notice.

🡪 Yes, we have checked for any missing reference numbers and ensured that the content aligns well with the main text. .

**Reviewer #1:**

1. The **methodology** is well-described, but considering **potential limitations like** **variability in patient anatomy** could strengthen the paper's depth.

Response

🡪 Thank you for the valuable feedback. Our study has potential limitations owing to the minimal anatomical changes in the organs used over the course of treatment. Therefore, further evaluation of our research methods by using other organs is necessary.

Modified [Line 357 - 361].

The prostate, rectum, and bladder considered in this study showed fewer morphological changes over time than the other organs. Our augmentation method learns these shape changes to generate images; therefore, minor morphological changes may make producing diverse images difficult. Additionally, we plan to recruit patients and focus on other organs to compare and validate our augmentation-based segmentation.

2. The discussion effectively underscores the significance of the segmentation method for prostate cancer radiation therapy. Briefly **discussing future directions** like **incorporating additional clinical variables** or **validating across diverse patient populations** would enrich the conclusion.

Response

🡪 Thank you for your insightful comment. As mentioned in the first response, we plan to recruit additional patients with different types of cancer and conduct evaluations using radiation therapy.

Modified [Line 357 - 361].

The prostate, rectum, and bladder considered in this study showed fewer morphological changes over time than the other organs. Our augmentation method learns these shape changes to generate images; therefore, minor morphological changes may make producing diverse images difficult. Additionally, we plan to recruit patients and focus on other organs to compare and validate our augmentation-based segmentation.

3. Highlighting the feasibility of automatic segmentation with two datasets per patient is commendable. However, **discussing encountered challenges and strategies to address them would provide a** **more balanced perspective**, enhancing the paper's contribution to adaptive radiotherapy.

Response

🡪 Thank you for your valuable comments. In this study, obtaining two sets of planning-quality CTs from patients was the most difficult. As CBCT is performed daily, it can be used instead of 2^nd^ set of planning-quality CT. Although the image quality of CBCT is poor, recent AI models have been developed to enhance the CBCT image quality to the level of planned CTs. We believe that daily ART will be possible if AI-based CBCT image quality upgrades are conducted in conjunction with our research. This is a limitation of the present study.

Modified [Line 348-351]

Our study has several limitations. We utilized simulated CT data and an additional unlabeled CT image taken during treatment. Additional CT scans are difficult to perform during the treatment for patients undergoing short-term treatments such as stereotactic body radiotherapy. If the improvement in the cone-beam CT images taken for setup verification is equivalent to the simulation CT images, additional CT scans can be eliminated.

4. While the methodology is comprehensive, a brief discussion on the evaluation of final segmentation models would enhance clarity. **Detailing the criteria used for selecting the best-performing model among 50 epochs** would ensure transparency and reproducibility.

Response

🡪 The explanation for the selection of the best-performance model was inadequate in the manuscript. Among the stored segmentation models, the model with the lowest Dice and cross-entropy loss on the validation dataset was selected as the best-performance model. We have added an explanation of this loss.

Before [Line 189 – 192]

For each epoch, the performance of the training model was evaluated with validation data, and the best-performing model among the 50 models from 50 epochs was selected as the final model for the patient; thus, 18 segmentation models were produced.

After [Line 191 – 193]

For 50 epochs, we saved the model at each epoch. Among the 50 models, the model with the lowest Dice and cross-entropy loss on the validation data was selected as the final model for the patient; thus, 18 segmentation models were produced.

5. The article needs **major improvements in English language** and there are many grammars and sentence error in this paper.

Response

The manuscript has been proofread by a professional English-language-editing agency.

**Reviewer #2:**

This paper describes a technique of creating individualized patient neural networks for organ segmentation in patients receiving radiation therapy for prostate cancer. It is well conceived and executed and well written. Its main strength is the utilization of data augmentation to demonstrate good segmentation performance with **very limited training data**. The technical aspects are clearly described and many examples are clearly presented. Its **main limitation is the limited number of patients (18) included**.

A few specific comments/questions:

1. Were all patients who presented for radiation treatment at Samsung Medical Center between November 18, 2019 and September 24, 2021 **included in the sample**? If there were **any patients excluded please explain why/exclusion criteria**. Potential bias in such a small sample may limit generalizability of findings.

Response

🡪 Not all patients were included in the sample. Only those with prostate cancer who agreed to undergo additional CT scans were included. Patients who did not consent to undergo additional CT scans were excluded.

Modified [Line 128 – 130]

We explained the research to adult male patients aged ≥18 years who underwent radiation treatment for prostate cancer between November 18, 2019, and September 24, 2021, and obtained signed consent forms from patients who expressed willingness to participate in additional CT scans.

2. In methods you state "if the loss decreased below 1000 and the generated image was similar to that of a human body, the DVFs were saved." Please clarify how "**similar to that of a human body" was defined - was this by manual visual inspection? If so, what was the time required to do this?**

Response

🡪 We agree that the sentence seems to be somewhat ambiguous. During the generation of the CT images using DVFs, when the loss exceeded 1000, the organs overlapped. When the loss dropped to below 1000, the three organs returned to their original positions. Therefore, when the loss of the augmentation method decreased to below 1000, the DVFs were saved and used as augmentation images. The time taken for the loss to decrease to below 1000 varied per patient and was generally approximately 3–10 min. This sentence has been revised accordingly.

Before [Line 163 – 166]

When the loss was large, ${CT}^{moved}$ generated via training did not appear to be similar to the image of the human body. After 250 iterations, which was equal to one epoch, if the loss decreased below 1000 and the generated image was similar to that of a human body, the DVFs were saved. As the iterations continued, nine more DVFs were saved such that ten DVFs were extracted for one image pair.

After [Line 163 – 168]

When the loss exceeded 1000, the organs in the generated ${CT}^{moved}$ overlapped, resulting in images that did not resemble the human anatomy. Therefore, we began saving the DVF as the loss decreased to below 1000. As the iterations continued, nine more DVFs were saved such that 10 DVFs were extracted for one image pair. Each epoch consisted of 250 iterations, and the training was terminated when 10 sets of DVFs with a loss below 1000 were completely saved. Approximately 3–10 min was required to save 10 sets of DVFs.

3. The **individual models were compared with a total model containing data for just 18 patients.** Is this a **big enough "total model"** or should further research be done to validate the individual model performance **against a larger total model**?

Response

🡪 The data from 18 patients were not sufficiently large for a comprehensive model. As shown in Table 4, in other studies, an average of 300 datasets has typically been used, with up to 876 datasets being used for training. For the comparison of the individual and total models using our planned VoxelMorph augmentation method, obtaining patient data that include two datasets from the same patient was challenging. Although when different data were used, compared with other studies, our results showed similar performance with significantly fewer datasets. Thus, our results for only 18 datasets demonstrate the performance similar to a much larger dataset. If additional datasets are obtained to validate a larger total model, the appropriate number of datasets required can be determined.

4. In your abstract conclusion you state "The proposed method can be clinically adopted for automatic prostate segmentation for adaptive radiation therapy." **Do you believe this model is ready for clinical use?** How would you propose it be adopted - to generate a "**first draft" radiation plan** which would be reviewed by a physician prior to radiation being given to the patient?

Response

🡪 We demonstrated that the performance of the proposed method is equivalent to those of models trained with a large dataset. For clinical applicability in patient treatment, a clinical routine in which a 2^nd^ CT is acquired needs to be established. As mentioned in the main text (lines348 -351), if we enhance the quality of daily CBCT images and use for our research method, auto segmentation for ART may be utilized clinically. In addition, any further performance improvement of the proposed model will be beneficial for clinical applications. Therefore, we have modified the abstract as follows:

modified [line 44 – 46, abstract conclusion]

We demonstrated the feasibility of automatic segmentation by employing individual patient datasets and image augmentation techniques. The proposed method has potential for clinical application in automatic prostate segmentation for ART.

5. Please proofread for minor errors, e.g. "Abstract" and periods inserted in the middle of sentences after references.

Response

🡪 The manuscript has been proofread by a professional English-language-editing agency.
